# Developing HIV-1 Protease Inhibitors through Stereospecific Reactions in Protein Crystals

**DOI:** 10.3390/molecules21111458

**Published:** 2016-10-31

**Authors:** Folasade M. Olajuyigbe, Nicola Demitri, Rita De Zorzi, Silvano Geremia

**Affiliations:** 1 Centre of Excellence in Biocrystallography, Department of Chemical and Pharmaceutical Sciences, University of Trieste, Trieste 34127, Italy; folajuyi@futa.edu.ng (F.M.O.); nicola.demitri@elettra.eu (N.D.); rdezorzi@units.it (R.D.Z.); 2 Department of Biochemistry, School of Sciences, Federal University of Technology Akure, P.M.B. 704, Akure 340252, Ondo State, Nigeria; 3 Elettra-Sincrotrone Trieste, S.S. 14 Km 163.5 in Area Science Park, Basovizza, Trieste 34149, Italy

**Keywords:** HIV-1 protease, epoxide-based inhibitor, reactions in crystals, X-ray crystallography, stereospecific inhibitors

## Abstract

Protease inhibitors are key components in the chemotherapy of HIV infection. However, the appearance of viral mutants routinely compromises their clinical efficacy, creating a constant need for new and more potent inhibitors. Recently, a new class of epoxide-based inhibitors of HIV-1 protease was investigated and the configuration of the epoxide carbons was demonstrated to play a crucial role in determining the binding affinity. Here we report the comparison between three crystal structures at near-atomic resolution of HIV-1 protease in complex with the epoxide-based inhibitor, revealing an in-situ epoxide ring opening triggered by a pH change in the mother solution of the crystal. Increased pH in the crystal allows a stereospecific nucleophile attack of an ammonia molecule onto an epoxide carbon, with formation of a new inhibitor containing amino-alcohol functions. The described experiments open a pathway for the development of new stereospecific protease inhibitors from a reactive lead compound.

## 1. Introduction

HIV-1 protease (PR) is an excellent target for antiretroviral therapy due to its crucial role in the life cycle of the human immunodeficiency virus (HIV). HIV-1 protease is a homodimer, with both chains participating in forming the active site of the enzyme. It processes the polyproteins into the structural proteins and enzymes during virion maturation [1]. Inhibitors of PR target the active site of PR to inhibit the activity of the enzyme, thus preventing cleavage of Gag and Gag-Pol polyproteins and resulting in production of non-infectious virus particles [1].

Different approaches have been employed in the design of PIs. Peptidomimetics reversibly inhibit the enzyme by competing with the substrate for the active site [2,3,4], while irreversible inhibitors form a covalent adduct with the target protease [5,6]. Some of these inhibitors were designed with the aid of X-ray crystallography [7,8], molecular docking [9,10], and click chemistry [11].

PIs-based therapies have had good success in improving the lives of infected people, especially when administered in the form of antiretroviral therapy (ART) which is based on a cocktail of two nucleoside reverse transcriptase inhibitors (NRTIs), together with a non-NRTI (NNRTI), a ritonavir-boosted protease inhibitor (PI) (atazanavir or darunavir) or an integrase strand-transfer inhibitor [12]. However, prolonged treatment regimens eventually face the challenge of drug resistance, an ever-escalating problem in AIDS management [13,14]. This has led to global research efforts directed towards overcoming drug resistance in HIV [15]. The development of irreversible inhibitors could represent a valuable tool in overcoming drug resistance considering that covalent binding might be less sensitive to mutations that reduce the binding affinity of the enzyme for inhibitors [5,6,16]. To date, a few epoxide-based inhibitors of PR have been reported that inhibit PR with high selectivity [16,17,18,19].

**Figure 1:**
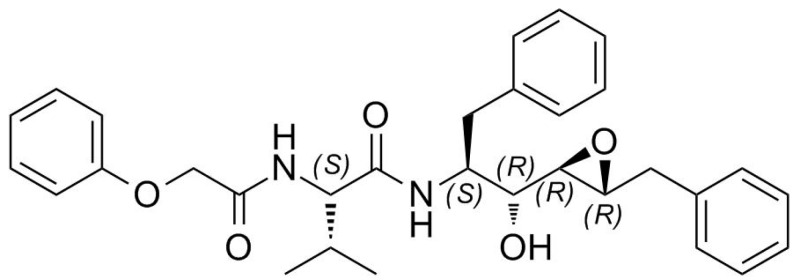
Structure of EPX inhibitor, (C_31_H_36_N_2_O_5_). MW = 516.63 g/mol.

It is widely acknowledged that structural information on protein targets complexed with inhibitors helps guide improved synthesis of lead compounds [20,21,22,23,24]. However, the direct use of protein crystals in making new ligands for a particular protein target by in-situ synthesis is just emerging [25]. Recently, we reported how the configuration of the epoxide carbons plays a crucial role in determining the binding affinity of an inhibitor [16]. PR was complexed with a new potent inhibitor containing an epoxy moiety within a Phe-Phe pseudodipeptide fragment (Figure 1).

**Figure 2:**
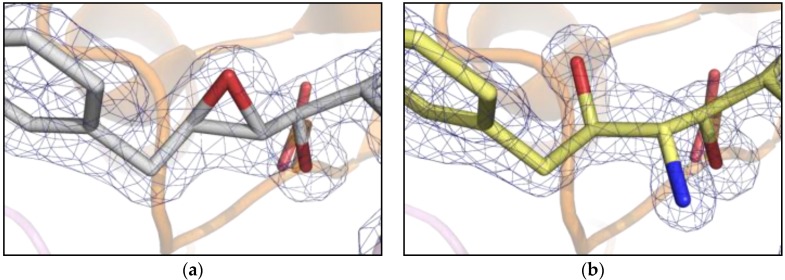
Comparison between electron density 2Fo-Fc maps (contour level 1.2 σ) near the active site of PR (cartoon). (**a**) pH 6.0, pseudopeptide epoxide inhibitor, EPX with unreacted/closed ring and catalytic aspartates in sticks; (**b**) pH 9.0, reacted pseudopeptide epoxide inhibitor showing open epoxide ring and formation of the amino diol derivative.

Analysis of the crystal structure of PR/EPX complex at 1.45 Å shows that the active site of PR is fully occupied by EPX in a single conformation with a surprisingly intact epoxide ring (Figure 2a). This result is in contradiction with the general behavior of epoxide-containing molecules that usually show high reactivity towards nucleophiles, leading to ring opening [16]. The presence of a reactive molecule trapped in the catalytic channel of PR opened the possibility to study solid state reactions. In particular, here we investigate triggering of an in-situ reaction in crystals of PR/EPX complex and suggest it as a possible route for developing stereospecific inhibitors of the HIV-1 protease.

## 2. Results

**Figure 3:**
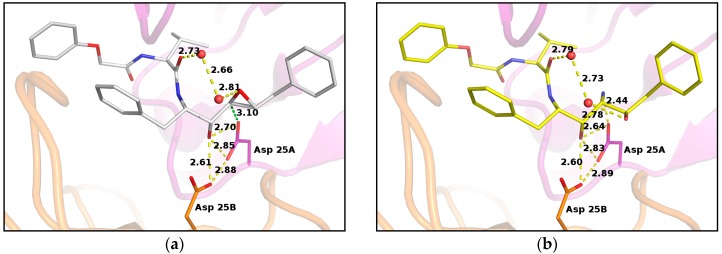
PR/EPX structures showing interactions with catalytic aspartates in PR/EPX before and after triggered reaction. (**a**) pH 6.0, PR/EPX structure showing interactions with catalytic aspartates. The carboxylate oxygen atom of the catalytic aspartate, in close contact to the oxirane carbon (green dashed line), forms an angle of 95° with the carbon–carbon bond of the ring; (**b**) pH 9.0, reacted pseudopeptide epoxide inhibitor produces a serinol derivative. The amino group forms a strong H-bond with a carboxylate oxygen atom of a catalytic aspartate. Interatomic distances are depicted as yellow dashed lines, except otherwise stated, with distance value in Angstrom.

Previous crystallographic studies on PR/EPX complex evidenced that the β-carboxylate group of the catalytic aspartate residue 25B is involved in hydrogen bonding with the hydroxyl group of the inhibitor, contributing to successfully stabilizing the inhibitor in the active site (Figure 3a) [16]. This carboxyl group is rather far from the epoxide carbon atoms. Conversely, the carboxyl group of the other aspartic acid 25A is in very close contact with one of the carbons of the oxirane ring. The distance between the two atoms is only 3.10 Å and the carboxylate oxygen atom is well aligned with the epoxide ring forming an angle of 95° with the carbon–carbon bond of the ring (Figure 3a). This value is close to the optimal trajectory calculated for the epoxide opening by Asp25 nucleophiles in HIV protease models by Mavri, who suggested that the deprotonated aspartate should attack the oxirane upon proton transfer to the leaving oxygen by the other protonated aspartic residue [26]. This proton transfer is clearly not possible in our case, as the epoxide oxygen is oriented upwards and far from the aspartic side chains. However, a water molecule was found at hydrogen-bond distance from the oxygen and could provide protons to assist the epoxide opening. This evidence suggested that the reaction did not occur at pH 6.0, as described by the reported crystal structure [16], because Asp25A was not available as a nucleophile in the catalytic site, since it was protonated.

The crystal structure of the PR/EPX complex with its intact oxirane ring (Figure 2a) suggested the possibility of investigating the ring opening reaction directly in the protein crystal. To promote the nucleophilic attack and the formation of an inhibitor covalently bound to PR, the deprotonation of the Asp25A was attempted by increasing the pH of drops containing the crystals. Thanks to the high degree of order present in the crystalline state of PR/EPX, as revealed by the electron density maps, the effect of triggering a reaction on the epoxide ring was investigated by a diffusion-based technique directly in the protein crystals.

In the soaking technique, the diffusion time is an important parameter to take into account for the diffusion of molecules into the crystal channels. Short diffusion times do not allow diffusion of molecules in the crystal, while prolonged diffusion periods put the crystal at risk of damage due to perturbation of the mother solution. The optimal time for diffusion was theoretically calculated taking into account the volume and shape of the crystals, the volume of channels accessible to the solvent present in the crystal structure, the concentration and size of the diffusing molecules [27]. The simulations of diffusion performed for small basic molecules such as OH^−^ or NH_3_, showed the possibility of saturating the crystals with the typical dimensions used in the experiments within few minutes. However, to avoid problems related to the kinetics of ring opening of the epoxide, in the final experiments, the system was allowed to equilibrate for a few hours before collecting X-ray diffraction data. The pH variation from 6.0 to 9.0 showed no damage to the PR/EPX crystals. On the contrary, severe crystal damage was observed lowering the pH below 6.0 in attempts to directly protonate the epoxide ring.

The X-ray structures determined to high resolution (in the range of 1.1 to 1.24 Å) by single crystal diffraction from synchrotron radiation show that the ring opening reaction was achieved in the PR/EPX crystals equilibrated with a pH 9.0 reservoir solution (Figure 2b). While the epoxide ring was found intact in the complex obtained at pH 6.0 (Figure 2a), at pH 9.0 the ring had opened. The electron density maps clearly proved that a stereospecific reaction had occurred at the oxirane ring with formation of new amino and alcohol functional groups. The chemical reaction of ring opening was achieved in PR/EPX crystals by diffusion of ammonia which acts as nucleophile.

In situ opening of epoxide ring led to the formation of a new stereospecific inhibitor containing amino-alcohol functions with a geometry defined by the catalytic site. The new functional groups contribute to the stabilization of EPX in the catalytic site of PR by providing a direct and strong H-bond interaction (2.44 Å) between the amino function of the inhibitor and a carboxylate oxygen atom of the catalytic aspartate Asp25A (Figure 3b).

**Figure 4:**
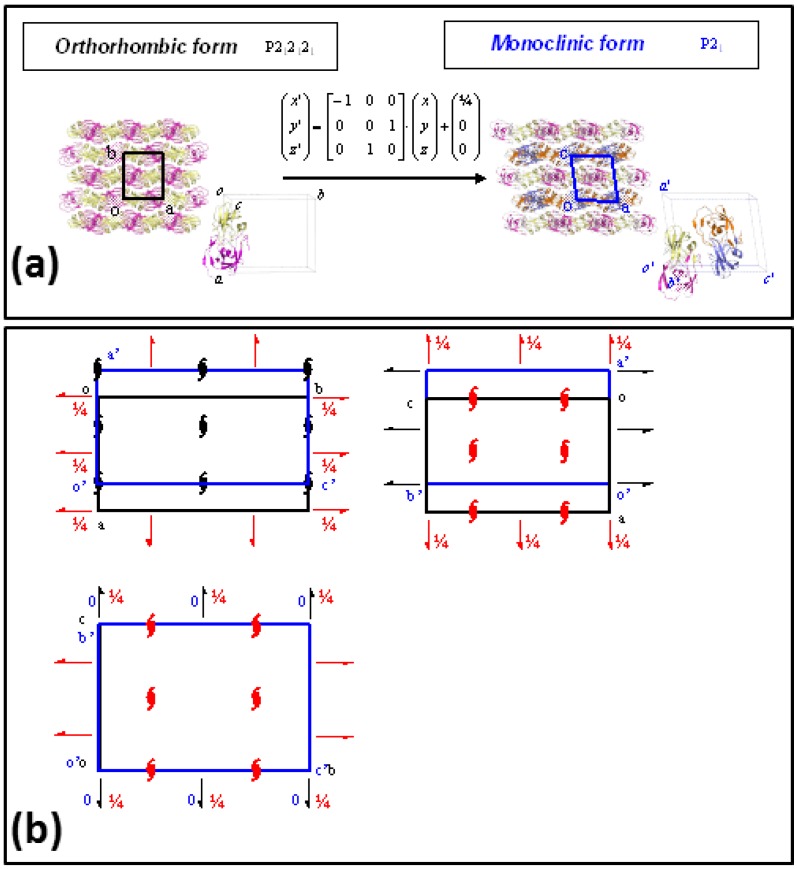
Group/subgroup relations in PR/EPX crystal forms. (**a**) Transformation of the asymmetric orthorhombic form (space group P2_1_2_1_2_1_) in that of monoclinic form (space group P2_1_). The transformation matrix and the translation vector of the atomic coordinates are also reported; (**b**) Superposition of the P2_1_2_1_2_1_ space-group diagram of the orthorhombic form (black) with the space-group diagram of the maximal non-isomorphic sub-groups P2_1_ of the monoclinic form (blue) shows the origin shift (1/4, 0, 0), the axes swapping (−a, c, b) and the missing symmetry operators (red symbols) to transform the lattice of orthorhombic form to that of monoclinic form.

Two different crystal forms, monoclinic and orthorhombic, related by a group/subgroup transformation (Figure 4), were characterized from crystals soaked in ammonia-citrate buffer, both of them showing a complete ring opening reaction, without crystallographic disorder in the catalytic site. Neither of the crystal lattices were disrupted or damaged in the reaction process, and the resolution limits of diffraction data, 1.24 Å for the monoclinic form and 1.12 Å for the orthorhombic, were even improved with respect to the initial data collected with the unreacted PR/EPX crystals (1.45 Å).

## 3. Discussion

**Figure 5:**
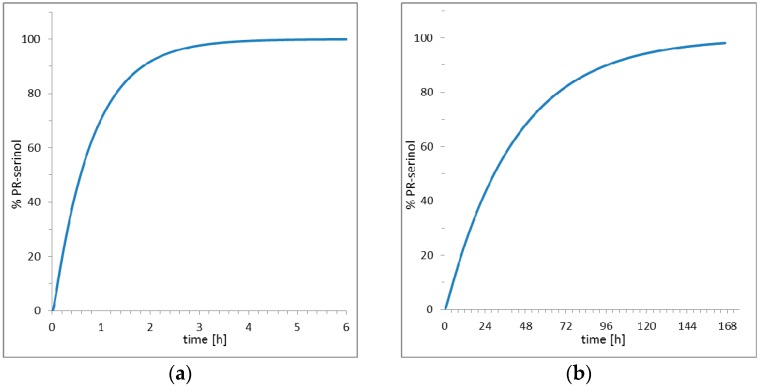
Simulation of diffusion, formation and complexation of amino diol derivative (**a**) hypothesis of reaction in PR/EPX crystal; (**b**) hypothesis of reaction in solution and diffusion into PR/EPX crystal.

The striking result obtained in this study is that the pH increase did not promote deprotonation of the catalytic aspartate with formation of irreversible covalent bond inhibitor. Rather, the nucleophilic ammonia molecule, produced by increasing the pH, diffused into the crystal and directly attacked the inhibitor and opened the epoxide ring. The opening of the epoxide ring led to the formation of a new inhibitor with two additional functional groups, hydroxyl and amine. Then, the nucleophilic attack of ammonia on oxirane ring of the inhibitor produced an amino diol derivative with common designation, serinol because it is a structural analogue to the amino acid serine [28]. The high-resolutions X-ray data collected after crystal perturbation suggests that the inhibitor reacted while bound to the active site of the enzyme, since an exchange with a preformed amino diol derivative with release of the epoxide inhibitor would require a large rearrangement of the protein domains [29,30] and a decrease in the order of the crystal. Such a hypothesis is also supported by simulation studies of the entire kinetic process which includes diffusion of small molecules into protein crystals, binding and release of inhibitors, and reaction with ammonia in or out of the crystal (Table S2). Two scenarios are presented in Figure 5 elaborated using COPASI [31] (a software application for simulation and analysis of biochemical networks and their dynamics) and the algorithm for simulation of diffusion processes in protein crystals earlier described [27]. With the hypothesis of reaction inside the crystal (Figure 5a), about 3 h are theoretically required to saturate the active site with amino diol derivative. On the contrary, the hypothesis which shows that the amino diol derivative is generated exclusively in the mother solution with the excess of the inhibitor and then it diffuses and replaces the EPX inhibitor in the protein crystal is presented in Figure 5b. The simulation (Figure 5b) shows a much slower process, with a very long duration, about one week, required to saturate the enzyme with the new inhibitor.

The acid-base behavior of amino acids is very important and plays crucial role in enzyme catalysis, substrate binding and protein structure [32]. Changing pH provides new insights into the mechanisms involved in protein activity [33]. In fact, Richman et al. reported that hydrogen exchange experiments promoted by altering pH in their study on the protein Nitrophorin 4 suggested backbone conformational fluctuations in the protein [34]. Regioselective ring-opening reactions of epoxides have been reported under basic conditions involving synthesis of biologically important intermediates [35]. In this work, ethyleneimine generated in situ from β-chloroethylamine was used as a nucleophile to open the epoxides in an aqueous environment. Another study demonstrated that primary amines undergo efficient ring opening reaction with epoxides [36]. The uniqueness of our results lies in the ability of triggering the ring-opening reaction of epoxide in a protein crystal. Chemical reactions within single crystals are rare due to the loss of crystallinity associated with atomic rearrangements [37]. However, the soaking technique was recently used to capture the reaction pathway in near-atomic-resolution crystal structures of PR [38,39]. In these protein structures, tetrahedral intermediates with short ionic hydrogen bonds to catalytic aspartates were observed.

It is likely that the ring opening reaction proceeded through a pseudo SN_2_ mechanism. The cleavage of the C-O bond and attack by the nucleophile, NH_3_, on the less sterically hindered carbon occurred in a single step. The water molecule, found at hydrogen-bond distance from the oxygen of the oxirane ring, assists the epoxide opening providing the required proton for formation of the hydroxyl group. This is similar to the reported mechanism of nucleophilic ring opening reaction of epoxide predicted using enzymatic model of HIV-1 protease and density functional theory methods [40].

From the stereochemical point of view, both oxirane carbon atoms of the EPX inhibitor, selected by PR, have absolute *R*-configuration (Figure 1). The carbon atoms of the epoxide ring opened to form hydroxyl and amine groups, the additional groups of the bound inhibitor, and conserved the *R*-configuration. It is well known that if the leaving group and the entering nucleophile have the same Cahn-Ingold-Prelog priority, such as in this case, the SN_2_ reactions should lead to an inversion of the absolute configuration of the reacting carbon atom. However, it should be noted that such incongruence is only apparent because the two alkyl groups of the R_1_R_2_CHNH_2_ stereogenic center switched their priority when the epoxide ring opened. It is interesting to observe that the ammonia attack occurred on the less sterically hindered but more interior oxirane carbon, occupying the protease P_1_ pocket. The newly formed stereospecific inhibitor with the amino function is particularly fascinating because it was recently suggested that introduction of a positive charge in an inhibitor at the active site of PR would create favorable charge-charge interactions with a deprotonated catalytic residue isolated from solvent [41].

The analysis of the hydrophobic and hydrogen bond interactions between PR and EPX (Figure S1) show many hydrophobic interactions between EPX and amino acid residues proline 81A, isoleucine 84A, glycine 27B, glycine 49A, valine 82B, proline 81B, and glycine 49B of PR, in addition to specific hydrogen bonds between EPX and the protein backbone of PR. In particular, EPX forms two hydrogen bonds with the carbonyl O atoms of 27A and 48A glycine residues, acting as acceptor, and a hydrogen bond with the NH of aspartate 29A, acting as donor. As recently reviewed, enhancing protein backbone binding in the active site is a fruitful strategy for combating drug-resistant HIV because the catalytic scaffold must be conserved to maintain functionality [42]. Interestingly, the interactions were very similar in the PR/EPX crystal structure with unreacted ring and PR/EPX crystal structure with triggered reaction on the epoxide ring (Figure S1). This is really striking because triggering of reaction in PR/EPX crystals did not disrupt the crystal lattice of PR. Actually, the diffraction resolution limit improved after increasing the pH.

Finally, two different crystal forms, orthorhombic and monoclinic, were obtained for PR/EPX complexes (Figure 4). The structural models obtained from the triggered reaction on the epoxide ring from both crystal forms overlap almost perfectly. In order to investigate the reason for this result, the group/subgroup relations and crystal packing of the two crystal forms were examined. In fact, the recognition of group-subgroup relations between space groups of different crystal structures can provide interesting insight into protein packing [43,44].

The two crystal forms of PR/EPX complexes reported in this paper, monoclinic and orthorhombic are related by a group-subgroup relation as shown in Figure 4a. The P2_1_2_1_2_1_ space group of the orthorhombic form is a minimal non-isomorphic supergroup of the P2_1_ space group of the monoclinic structure. Space-group and packing diagrams are particularly useful to find the possible relationship between these two structures (Figure 4b) which show very similar intermolecular positions and contacts. This analysis confirms the evidence that the two crystal forms have very similar crystal packing, therefore the analogous behavior with respect to the reaction within crystal of these two crystal forms is not surprising.

In conclusion, this study has unveiled a new possibility for reaction initiation by diffusion based technique. Triggering of reactions in crystals of PR complexes confirms that the crystalline state is not a barrier and reactions can be completed in protein crystals. Thanks to their high solvent content that permits some molecular flexibility, protein crystals can be exploited to gain a better understanding of their function in molecular terms. The reaction mechanism in PR/EPX crystals might be the starting point for developing stereospecific inhibitors of PR and potential prodrugs with a pH-triggered activation profile. In particular, it is an example of a reactive lead compound that can produce in situ a stereospecific inhibitor. These results should be valuable in the design of future generations of stereospecific inhibitors of PR and epoxide based antiviral drugs capable of combating the escalating problem of drug resistance.

## 4. Materials and Methods

### 4.1. Expression, Purification and Refolding of HIV-1 Protease

HIV-1 protease, PR (Genbank HIVHXB2CG) was stabilized by five mutations: Q7K, L33I, L63I to minimize the autoproteolysis, and C67A and C95A to prevent cysteine-thiol oxidation/aggregation by the formation of disulfide bonds. The PR was expressed in *Escherichia coli* BL21-Gold (DE3)pLysS competent cells. The expressed PR was purified from inclusion bodies by a modified method of Louis and co-workers [45]. Using consolidated refolding and crystallization protocols [23], the protein (1–2 mg·mL^−1^) was preincubated with a 5-fold molar excess of the inhibitor, EPX.

### 4.2. Crystallization and Triggering of Reactions in Crystals of PR/EPX

The crystallization drops were formed using 1 µL of reservoir solution (0.25 M sodium citrate pH 6.0, 10% DMSO, and 40%–60% saturated ammonium sulfate) and 1 µL of a solution of protein with inhibitor. Crystals of typical dimension 0.4 mm × 0.2 mm × 0.3 mm were grown at 20 °C by vapor diffusion using the hanging drop method. X-ray structures of PR/EPX crystals showed non-covalent binding of EPX with closed ring of the epoxide in the active site of PR [16]. Successive experiments were performed on crystals of PR/EPX grown at pH 6.0. The pH of drops containing crystals was varied by vapor diffusion through altering pH of the reservoir solutions in the range of pH 2.0 to 12.0. The minimum time of diffusion of small ions or molecules such as hydroxonium, hydroxyl or ammonia in the crystal was evaluated with an algorithm earlier described [27] for the simulation of diffusion of small molecules inside protein crystals using the COPASI program [31]. In final experiments, the crystals in drops were allowed to equilibrate for about 6 h against a reservoir added with saturated ammonium sulfate at pH 10.0, before collecting diffraction data. The final pH of the reservoir solution was later measured to be 9.0.

### 4.3. Crystallographic Analysis

Data were collected for PR/EPX crystals on the XRD1 diffraction beam-line of the ELETTRA Synchrotron, Trieste, Italy (Table S1). Crystals were cryoprotected with 20% glycerol, mounted on a nylon loop and flash-frozen in liquid nitrogen. Data collections were performed at 100 K on a MAR-CCD detector (Rayonix, L.L.C, Evanston, IL, USA) and were processed using Mosflm and CCP4 suite [46,47]. Structure refinement was conducted using REFMAC [48] and Coot [49], starting from isomorphic crystal structures (PDB code: 2NMZ). Alternative conformations for residues were modeled where appropriate, and water molecules were inserted in the model based on peaks greater than 3 σ in F_obs_-F_calc_ maps. The ions and solvent molecules were identified by the shape of the 2F_o_-F_c_ electron density map and their inter-atomic distances. All figures were made using PyMol [50] and coordinates for the structures have been deposited in Protein Data Bank. PDB codes: 3TOF for PR/EPX structure at pH 6.0, 3TOG and 3TOH for PR/EPX structures at pH 9.0.

## Supplementary Materials

Supplementary materials can be accessed at: http://www.mdpi.com/1420-3049/21/11/1458/s1. Table S1: X-ray Data collection and Refinement Statistics, Figure S1: LIGPLOT representations of the interactions between EPX and PR (Figure S1), Table S2: Parameters used for COPASI simulation of diffusion, formation and complexation of serinol derivative in PR/EPX crystals. Atomic coordinates of protein-inhibitor complexes are deposited with the Protein Data Bank (PDB), PDB codes: 3TOF for PR/EPX structure at pH 6.0, 3TOG and 3TOH for PR/EPX structures at pH 9.0.
