# Supplementary material for: Developing HIV-1 Protease Inhibitors through Stereospecific Reactions in Protein Crystals
Source: Molecules. 2016 Oct 31;21(11):1458. doi: 10.3390/molecules21111458 (PMC6273989; doi:10.3390/molecules21111458)
Supplement: Supplementary file 1 [file molecules-21-01458-s001.pdf]

Supplementary Materials: Developing HIV-1 Protease Inhibitors through Stereospecific Reactions in Protein Crystals

Folasade M. Olajuyigbe, Nicola Demitri, Rita De Zorzi and Silvano Geremia

Table S1. X-ray Data collection and Refinement Statistics.

PR/EPX Complexes			
**Reservoir pH**	**pH 6**	**pH 9**	**pH 9**
PDB ID	3TOF	3TOH	3TOG
Crystal Form	Orthorhombic	Orthorhombic	Monoclinic
Space group	$P2_12_12_1$	$P2_12_12_1$	$P2_1$ (β = 99°)
a [Å]	51.287 (4)	51.266 (5)	51.195 (2)
b [Å]	58.395 (7)	58.382 (9)	62.143 (7)
c [Å]	61.072 (2)	61.304 (11)	58.768 (10)
Volume [Å^3]	182,900	183,480	184,660
Maximum resolution (Å)	1.45	1.11	1.24
Reflections (I/σ>2)	30,233 (23,666)	66,936 (52,396)	98,017 (76,725)
R_free reflections	1512	3347	4901
Restrains	20,916	20,952	40,620
Parameters	15,687	15,714	30,465
R_factor (I/σ>2) %	18 (17)	18 (17)	22 (20)
R_free (%)	24.6	21.1	26.4
Final model			
Protein atoms	1512	1512	3024
Inhibitor atoms	38	39	78
Water molecules	177	183	271
Other atoms	16	12	12
RMS Deviation			
Bond lengths (Å)	0.023	0.012	0.023
Bond angles (Å)	0.024	0.017	0.021
B-factor (Å^2)			
Protein main chain	11.0	9.5	12.1
Protein side chains	14.6	13.0	15.7
Inhibitor	19.5	17.1	18.9
Water molecules	25.1	22.4	23.4
Other molecules	20.7	16.4	18.6

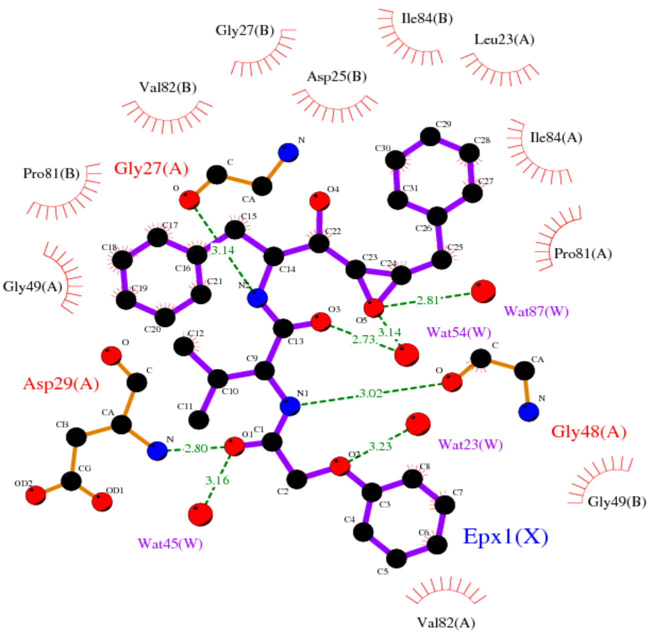

(**A**)

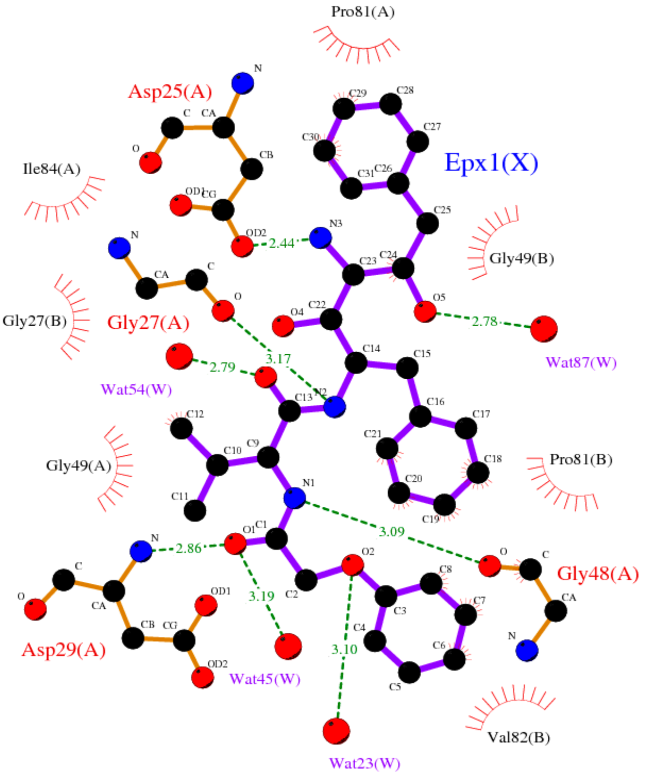

(**B**)

Figure S1. (**A**) LIGPLOT of hydrophobic and hydrogen bond interactions between EPX and PR residues in PR/EPX with closed/unreacted epoxide ring; (**B**) LIGPLOT of hydrophobic and hydrogen bond interactions between EPX and PR residues in PR/EPX with triggered reaction on epoxide ring.

Table S2. Parameters used for Copasi simulation of diffusion, formation and complexation of serinol (SER) derivative in PR-EPX crystals (Figure 3 of manuscript). "Scenario 2" is equivalent to "Scenario 1" with reaction R7 omitted (formation of SER in PR/EPX crystal).

**Compartments**		
**Compartments**	**Solution (sol)**	**Crystal (cry)** [a]
Volume (cm^3)	1.0×10^{-3}	2.4×10^{-5}

[a] Assuming a typical crystal size of (0.4, 0.3, 0.2) mm^3, corresponding to a A/Δx ratio of 0.41 cm [1], for the pH 6.0 orthorhombic crystal form reported in Table S1 (PDB_{ID} 3TOF).

**Molecular properties**			
**Molecule**	**NH_3**	**EPX**	**SER**
Cell accessible Volume Φ (fraction)	0.121	0.033	0.033
D (cm^2/s) [b]	2.9×10^{-5}	3.9×10^{-6}	3.9×10^{-6}
k_D'(cm^3/s) [c]	1.7×10^{-7}	1.7×10^{-9}	1.7×10^{-9}

[b] Calculated by HYDRO [2]. [c] $k_D' = D\,\Phi^2 A/\Delta x$ [1].

**Concentrations**						
**Molecule**	**NH_3**	**EPX**	**SER**	**PR**	**PR/EPX**	**PR/SER**
Solution concentration (mM)	4×10^3	0.38	0			
Crystal concentration (mM)	0	0.38	0	0	3.6	0

**Diffusion Model Reactions**				
**Label**	**Expression**	**Type**	**k_1**	**k_{-1}**
R1	EPX(sol) = EPX(cry)	M.A.(rev.) [d]	1.7×10^{-6} µL/s	1.7×10^{-6} µL/s
R2	PR(cry) + EPX(cry) = PR/EPX(cry)	M.A.(rev.)	5.8×10^2(mM s)$^{-1}$ [e]	8.1×10^{-4} s^{-1} [e]
R3	SER(sol) = SER(cry)	M.A.(rev.)	1.7×10^{-6} µL/s	1.7×10^{-6} µL/s
R4	PR(cry) + SER(cry) = PR/SER(cry)	M.A.(rev.)	5.8×10^2(mM s)$^{-1}$ [e]	8.1×10^{-4} s^{-1} [e]
R5	EPX(sol) + NH_3(sol) -> SER(sol)	M.A.(irrev.) [d]	1.0×10^{-7}(mM s)$^{-1}$ [f]	
R6	NH_3(sol) = NH_3(cry)	M.A.(rev.)	1.7×10^{-4} µL/s	1.7×10^{-4} µL/s
R7	PR/EPX(cry) + NH_3(cry) -> PR/SER(cry)	M.A.(irrev.)	1.0×10^{-7}(mM s)$^{-1}$ [f]	

[d] COPASI Mass Action (M.A.) reversible or irreversible reaction [3]. [e] Assuming same values reported for Ritonavir inhibitor in [4]. [f] Evaluated from analogue oxirane ring opening reaction reported in [5].

References

1. Geremia, S.; Campagnolo, M.; Demitri, N.; Johnson, L.N. Simulation of diffusion time of small molecules in protein crystals. *Structure* **2006**, *14*, 393–400.
2. Garcia de la Torre, J.; Navarro, S.; Lopez Martinez, M.C.; Diaz, F.G.; Lopez Cascales, J. HYDRO. A computer software for the prediction of hydrodynamic properties of macromolecules. *Biophys. J.* **1994**, *67*, 530–531.
3. Hoops, S.; Sahle, S.; Gauges, R.; Lee, C.; Pahle, J.; Simus, N.; Singhal, M.; Xu, L.; Mendes, P.; Kummer, U. COPASI—A Complex PAthway SImulator. *Bioinformatics* **2006**, *22*, 3067–3074.
4. Dierynck, I.; De Wit, M.; Emmanuel, G.; Keuleers, I.; Vandersmissen, J.; Hallenberger, S.; Hertogs, K. Binding Kinetics of Darunavir to Human Immunodeficiency Virus Type 1 Protease Explain the Potent Antiviral Activity and High Genetic Barrier. *J. Virol.* **2007**, *81*, 13845–1385.
5. Stropoli, S.J.; Elrod, M.J. Assessing the Potential for the Reactions of Epoxides with Amines on Secondary Organic Aerosol Particles. *J. Phys. Chem. A* **2015**, *119*, 10181–10189.
